# Efficient electrocatalytic oxygen reduction reaction of thermally optimized carbon black supported zeolitic imidazolate framework nanocrystals under low-temperature†

**DOI:** 10.1039/d3ra07754c

**Published:** 2023-11-24

**Authors:** Jinyi Chen, Jian Guo, Hong Zhang, Dan J. L. Brett, Srinivas Gadipelli

**Affiliations:** a College of Physics, Sichuan University Chengdu 610064 China; b Electrochemical Innovation Lab, Department of Chemical Engineering, University College London London WC1E 7JE UK s.gadipelli@ucl.ac.uk

## Abstract

Turning commercially available low-cost conducting carbon black materials into functional electrocatalytic electrode media using simple surface chemical modification is a highly attractive approach. This study reports on remarkably enhanced oxygen electrocatalytic activity of commercially available Ketjenblack (KB) by growing a non-precious cobalt metal-based zeolitic-imidazolate framework (ZIF-67) at room temperature in methanol solution followed by a mild thermolysis. The resulting Co@CoO_*x*_ nanoparticle decorated nitrogen-doped KB derived from the optimized ZIF-67 : KB weight ratio of hybrid samples at 500–600 °C shows high performance for the oxygen reduction reaction (ORR) with impressive *E*_onset_ and *E*_1/2_ values of ∼0.90 and ∼0.83 V (*vs.* RHE), respectively in 0.1 M KOH electrolyte. Such ORR activity is comparable to, or better than many metal@metal-oxide-carbon based electrocatalysts synthesized under elevated carbothermal temperatures and using multicomponent/multistep chemical modification conditions. Therefore, a simple electrocatalyst design reported in this work is an efficient synthesis route that not only utilises earth-abundant carbon black but also comprises scalable room temperature synthesized ZIF-67 following mild thermolysis conditions under 600 °C.

## Abstract 2

Co@CoO_*x*_ nanoparticle decorated nitrogen-doped carbon derived from zeolitic-imidazolate framework (ZIF-67)@Ketjenblack under mild thermolysis condition delivered impressive ORR activity (*E*_onset_ = ∼0.90 V; *E*_1/2_ = ∼0.83 V) in alkaline electrolyte.

## Graphical abstract

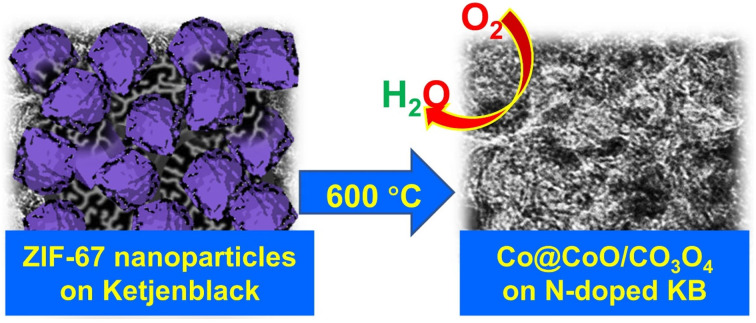

## Introduction

Simple and scalable design routes for electrocatalysts are required for the widespread implementation of electrochemical-based clean energy technologies that rely on catalytic processes with efficient conversion of molecular reactant species.^1^ For example, the performance, efficiency and life-span of next-generation high-energy rechargeable metal-air batteries and hydrogen-based proton-exchange fuel cells depend on the reduction and oxidation of molecular oxygen and hydrogen feedstocks. Here, the oxygen reduction reaction (ORR) is one of the important half-cell reactions and is considered the performance determining reaction.^1–10^ Platinum metal nanoparticles dispersed on carbon black materials (usually denoted as Pt/C) are typical active electrocatalysts.^1^ However, their high cost factor and unsatisfactory catalytic durability hinder the widespread implementation of electrochemical technologies. Therefore, the development of high-performing non-precious electrocatalyst materials has attracted huge research attention.

Numerous design/synthesis routes have been discussed for generating functional electrocatalyst materials.^1–36^ Among these, carbothermal synthesis of metal-coordinated small molecular or polymeric networks have shown promising electrocatalytic activities. Pre-synthesized porous networks, such as metal–organic frameworks (MOFs) are often utilised precursors with the choice of enriched metal centres and ligand/linker carbon network with functional heteroatom groups, which can enhance the electrophilicity and heterogeneity of the metal-carbon network and thus promote the adsorption/desorption of reactants/intermediates during the catalytic process.^1,3–15,18,22–24^ MOFs, such as ZIFs comprising highly accessible porosity and surface area along with metal and nitrogen elements have delivered superior functional electrocatalysts in the form of metal–nitrogen–carbon (usually denoted as M–N–C) or metal/metal-oxide nanoparticle embedded N–C matrix (MO_*x*_–N–C or M@MO_*x*_–N–C). For example, the carbothermal modulation of Co metal based MOFs/ZIFs can generate Co–N–C or Co@Co_3_O_4_ and/or Co@CoO_*x*_ nanoparticles embedded N–C materials.^3–5,7,10–13,15,18,22,23,27–36^ However, the high-temperature carbothermal modification process, commonly carried out between 800–1000 °C for a few hours, along with significant ligand volatility and metal-centres agglomeration, limits their full potential of scalable synthesis and efficient electrocatalytic performance. Attempts to use low-temperature carbothermal synthesis resulted in unsatisfactory electrochemical performance due to poor electrical conductivity of carbon network.^4,5,11,12,22,23^

Pre-fabricated conducting carbon supports have been used to support polymer, MOF or ZIF structures and exploited for their electrocatalytic activities under low carbothermal modulation. For example, reduced graphene-oxide (rGO),^4,15,29,31^ carbon nanotube (CNT),^9,18,21,27^ carbon cloth/paper (CC/CP), carbon nanofiber (CNF),^2,10,13,19,32,33^ small metal-coordinated molecules/polymers or MOFs-derived carbon (MDC) templated and/or ordered mesoporous carbon (OMC)^2–4,6,7,9,11,16,22,23,27–36^ have been used. However, such pre-synthesized carbon supports offer no commercial/scalable benefits as they require complex and prolonged chemical modifications. Here, it is worth mentioning that rGO synthesis involves high volumes of concentrated acids/metal salt-related oxidation processes followed by prolonged washings and reduction under reducing chemical agents or thermal treatments.^15^ Similarly, CNTs, MDCs or OMCs requires chemical vapour deposition, and pre-assembled templates and/or molecular forms under high-temperature carbonization conditions. Although some interesting electrocatalytic activities have been achieved with multicomponent materials comprising combinations of metal-centres as well as heteroatom dopants, the overall functional materials development requires a simple design and synthesis route.

Considering these factors, the present work utilises easily accessible and commercially available low-cost and electrically conducting carbon black, Ketjenblack EC-300J (KB), which is widely used in battery, fuel cell and supercapacitor electrode structures. It is also classified as a non-hazardous solid material. KB offers a high specific surface area of approximately 800 m^2^ g^−1^ and its unique graphitic morphology and porous structure with a density of 0.125–0.145 g cm^−3^ makes it one of the preferred materials for MOF crystal growth and electrochemical catalytic applications, as presented in this work. As-purchased KB powder was used directly in the methanol solution containing MOF-precursor constituents for the synthesis of cobalt-based ZIF (ZIF-67) nanoparticles on its surface (named *x*ZKB, where *x* refers to the weight percentage of ZIF-67 in the ZKB composite, *e.g.*, 60ZKB contains about 60 wt% of ZIF-67, see ESI for experimental section and Table S1†). Interestingly, these as-produced *x*ZKB samples treated under mild-temperature conditions of 500–600 °C showed impressive electrocatalytic ORR activity 0.1 M KOH electrolyte. Compared to thermolyzed samples of ZIF-67 and KB-controlled, the thermally optimized 60ZKB sample at 600 °C (named 60ZKB-600) exhibited highly improved ORR performance with competitive onset and half-wave potential (*E*_onset_ and *E*_1/2_, respectively) values of ∼0.90 and ∼0.83 V (*vs.* RHE), which are comparable or better than many carbon-based Co–N–C and CoO_*x*_–N–C or Co@CoO_*x*_–N–C electrocatalysts synthesized under higher thermolysis and prolonged chemical modification conditions.^1–24,27–34^ Structural and electrochemical characterisations by powder X-ray diffraction (XRD), X-ray photoelectron spectroscopy (XPS), scanning electron microscopy (SEM), transmission electron microscopy (TEM), cyclic voltammetry (CV) and linear sweep voltammetry (LSV) of the samples reveal the fine-dispersion of Co@CoO/Co_3_O_4_ nanoparticles (∼5 nm size) in N-doped KB support. Thus, results and analysis presented in this work indicate that low-cost and abundantly available carbon black materials can be turned into highly active electrocatalyst materials by surface grown MOFs and their mild carbothermal optimization at or below 600 °C.

For this, initially, 80ZKB sample was synthesized as it contains about 20 wt% of KB, which is a typical carbon black additive amount in the electrode materials applied for various electrochemical energy conversion and storage applications. From this, the acceptable ORR performance and suitable thermolysis temperature was obtained by comparatively screening the thermolyzed 80ZKB and ZIF-67 samples, produced at different temperatures of 500, 600 and 700 °C. Building on this understanding, the ORR performance was further optimized from different composition of *x*ZKB samples, following thermolysis under 700 °C. The structural-performance insights of best ORR performing catalyst with respect to literature reported data was presented to support our facile chemical modulation of ZKB samples under relatively low-temperature.

## Results and discussion

**Fig. 1:**
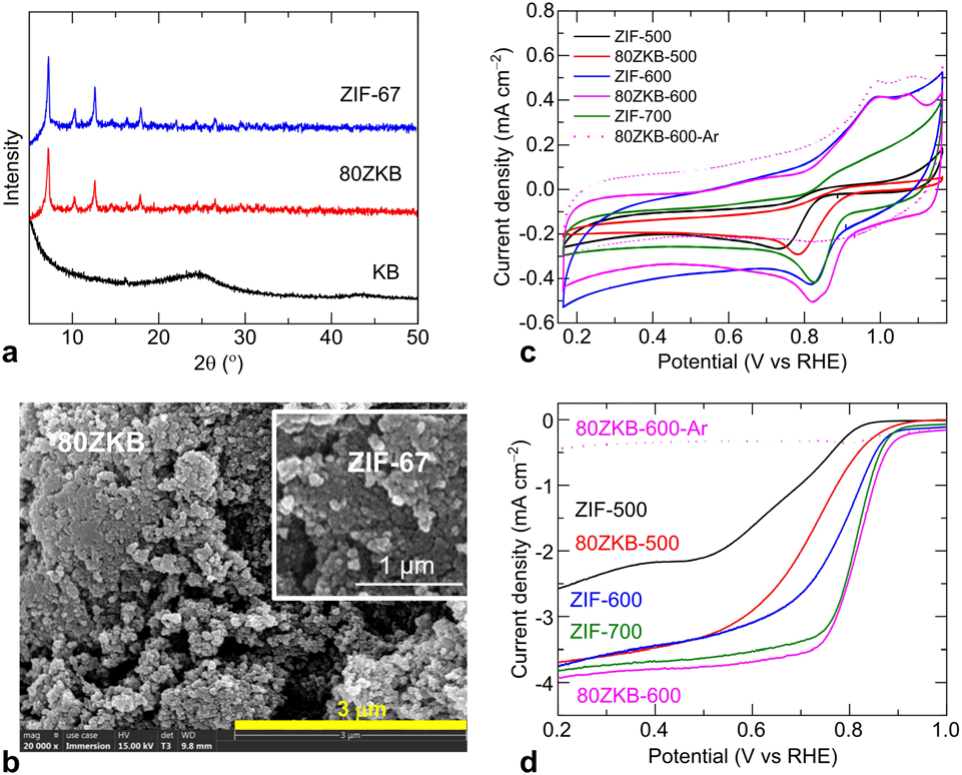
(a) Powder XRD patterns of KB, ZIF-67 and 80ZKB samples. (b) SEM images of 80ZKB and ZIF-67 (inset). (c and d) CV and LSV curves of 80ZKB and ZIF-67 samples thermolyzed at 500, 600 and 700 °C. Dotted line data were measured under Ar-purged electrolyte.

KB was used as-purchased for the synthesis. Initially, before going to full range composition of *x*ZKB samples synthesis, the optimum thermolysis temperature and ORR performance evolution in the *x*ZKB samples, with respect to ZIF-67, were identified by synthesizing 80ZKB sample. 80ZKB was selected as it comprises 20 wt% KB, which is a typical carbon black additive in the electrode structures used in electrochemical applications. ZIF-67 and 80ZKB samples were obtained by room-temperature stirring of methanolic solution containing precursors, according to the previously reported works.^8,14,15^ Powder XRD patterns and SEM images shown in Fig. 1a and b confirms the nanocrystalline ZIF-67 particles with ∼50 nm size. These small sized nanoparticles often appear aggregated and do not show a typical dodecahedral-shaped structure, which usually develops for the particle size of about 100 nm or more.^8,14,15,22–26^ These as-synthesized ZIF-67 and 80ZKB samples were thermolyzed under mild-temperatures at 500, 600 and 700 °C (*e.g.*, ZIF-67 thermolyzed at 500 °C is named as ZIF-500).

The electrocatalytic ORR activity of these samples was evaluated in a typical three-electrode system. Fig. 1c and d shows the ORR activity evolution by CV and LSV of the samples. Here, evolution of the ORR activity in the samples can be seen from the characteristic change of CV and LSV curves. The development of a cathodic peak in the CV curves and/or a flat or wide-slope-like to step-like behaviour of the LSV curves with increase in thermolysis temperature suggests increased ORR activity.^1^ Note that the samples tested under N_2_-purged electrolyte show no such features in the CV and LSV curves (*e.g.*, the dotted line data of the 80ZKB-600 sample). Compared to thermolyzed ZIF-67 samples, a rapidly enhanced ORR activity is seen in the 80ZKB samples thermolyzed at 500 and 600 °C. For example, 80ZKB-600 sample ORR activity is even outperforming ZIF-700 by offering more positive *E*_onset_ (∼0.9 V) and *E*_1/2_ (∼0.82 V) values. Literature data shows that ZIF-67 or relevant MOF or metal-coordinated molecular structures required to carbonise at greater than 700 °C,^1–7,9–12,15–19,22–24,27–34^ however, they show inferior ORR activities than the 80ZKB-600 sample; this is linked to significant graphitization of ligand carbon, which become hydrophobic, along with severe agglomeration of Co(0) nanoparticles. For example, ZIF-67 carbonized at 800 °C under 5%H_2_/Ar atmosphere displays inferior *E*_onset_, *E*_1/2_ and limiting-current density (*J*_L_) values of 0.86 V, 0.78 V, and 4.1 mA cm^−2^, respectively.^4^ Likewise, *E*_1/2_ of 0.68 to 0.8 V with *J*_L_ of 2.8 to 3.9 mA cm^−2^ was observed for the Co–N–C materials derived directly from pyrolyzed ZIF-67 or other Co containing polymeric precursors.^1,5,7,9,10,23,27–30^

**Fig. 2:**
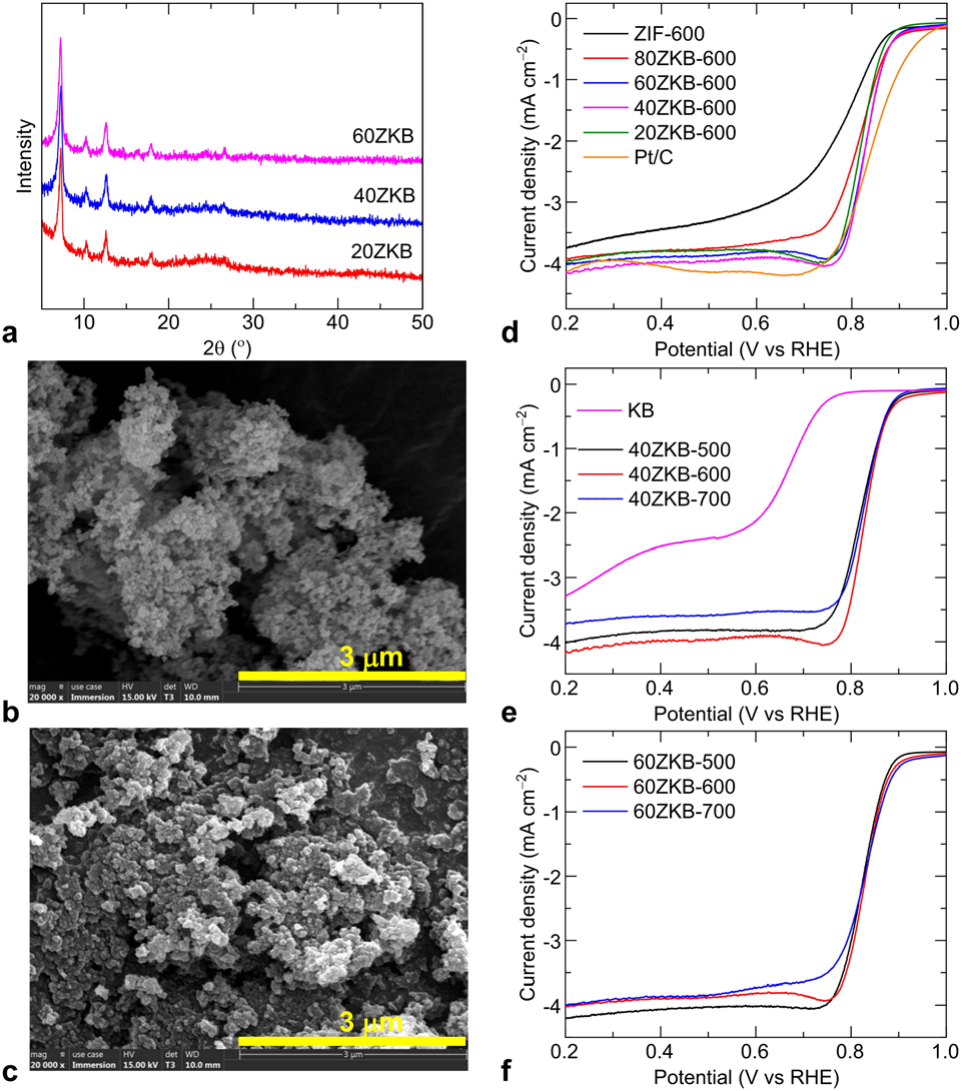
(a) Powder XRD patterns of *x*ZKB samples. (b and c) SEM images of 20ZKB (b) and 60ZKB samples (c). (d) LSV curves of *x*ZKB-600, ZIF-600 and reference Pt(20 wt%)/C samples. (e and f) Comparative LSV curves of thermolyzed 40ZKB and 60ZKB samples at three different temperatures of 500, 600 and 700 °C.

Motivated by the high ORR activity in the 80ZKB-600, the amount of ZIF-67 nanophase grown on KB was further varied to achieve optimal ORR performance. For this, more *x*ZKB samples forming 20, 40 and 60 wt% of ZIF-67 on KB were synthesized. As illustrated in Fig. 2a–c, the XRD patterns and SEM images reflect an increased amount of ZIF-67 nanoparticle growth for the samples of 20ZKB to 60ZKB. Next, the ORR performance of these samples thermolyzed at 600 °C, displayed in Fig. 2d and S1–S4,† reveal the impressive composition-dependent ORR activity trend. A remarkably improved ORR performance can be in the 60ZKB-600 sample by offering high positive *E*_onset_ and *E*_1/2_ values close to 0.90 and 0.83 V compared to 0.87 V and 0.77 V, respectively from ZIF-600. Furthermore, the comparative LSV curves of 20ZKB, 40ZKB and 60ZKB samples modulated under low temperatures at 500 and 600 °C show optimal performance compared to samples carbonised at 700 °C (Fig. 2e, f and S2 and S3†). Here, among the samples tested, 60ZKB-600 shows best ORR performance and its *E*_1/2_ and *J*_L_ values are close to reference Pt/C sample under same experimental setup. KB alone shows a negligible activity. Moreover, the literature data on the Co_3_O_4_–N–C based nanostructures show inferior ORR activity (Table S2†).^1,4,7,11,13,18–21,23,27,31–34^ For example, Co_3_O_4_@N-doped KB synthesized by carbonizing pre-oxidised KB, after treating with concentrated HNO_3_, and melamine mixture at 700 °C following hydrothermal route delivered *E*_onset_ and *E*_1/2_ values between 0.81–0.87 V and 0.73–0.82 V, respectively.^20^ Likewise, Co_3_O_4_–N–C obtained by carbonization of freeze-dried cobalt-phthalocyanine with alkali solution at temperatures of 700–1000 °C offered *E*_onset_ and *E*_1/2_ of 0.85 V and 0.77 V, along with *J*_L_ of 3.9 mA cm^−2^.^11^ The Co_3_O_4_, Mn_3_O_4_ and bimetallic Mn_*x*_Co_3−*x*_O_4_ nanoparticles decorated N-doped CNT also offered *E*_onset_ of ∼0.82 V, *E*_1/2_ of 0.73–0.78 V and *J*_L_ of ∼4.0 mA cm^−2^; these samples were synthesized by utilising pre-oxidised multi-walled CNT with concentrated HNO_3_ under refluxed conditions following solvothermal growth of oxide nanoparticles or air oxidation.^18,21^ All these results indicate that electrochemical activity is directly enabled by the thermally generated active phase structure of 60ZKB.

**Fig. 3:**
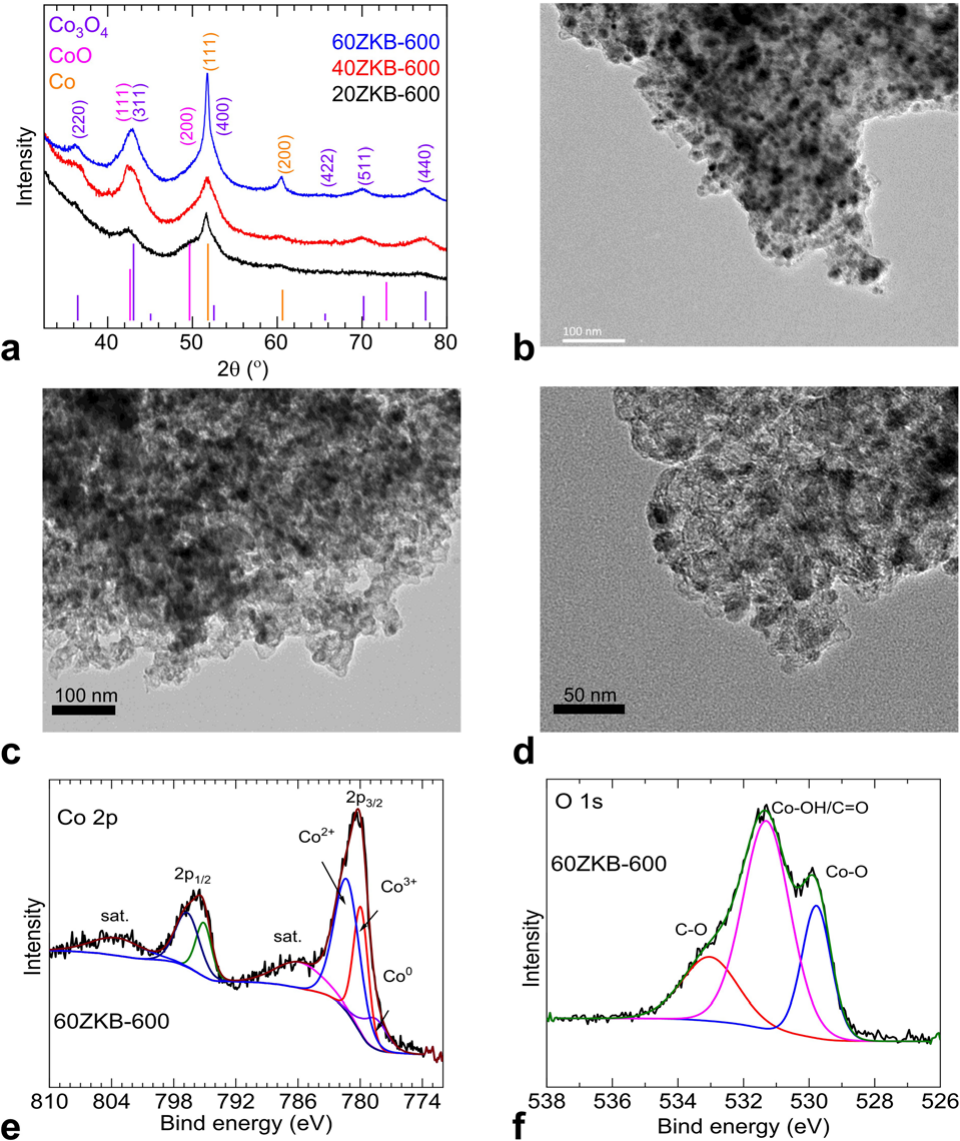
(a) Powder XRD patterns of *x*ZKB-600 samples. (b–d) TEM images of ZIF-600 (b) and 60ZKB-600 ((c and d) at different magnification) samples. (e and f) High-resolution XPS Co 2p and O 1s spectra with deconvoluted peak fittings for 60ZKB-600 sample.

Thermolyzed ZIF-67 and *x*ZKB samples at 600 °C were probed by powder XRD, TEM and XPS analytical techniques. As presented in Fig. 3a, XRD patterns show weak broad peaks relevant to a nanocrystalline Co_3_O_4_ main phase, belonged to cubic spinel structure (JCPDS card no. 43-1003) along with a minor fraction of CoO (face-centred cubic (fcc) structure, JCPDS card no. 43-1004) and Co metallic fcc structure, JCPDS card no. 15-0806). For example, CoO phase can be seen with a merged and shoulder peaks with Co_3_O_4_ and metallic Co phases, respectively at close to 42.2° and 50° of 2-theta values, as marked by the relevant (*hkl*) assignments. From this point, to gain more structural insights of the best ORR performing 60ZKB-600 catalyst was further examined by TEM and XPS techniques. As shown in Fig. 3b–d and S5,† the TEM micrographs reveal a nanoparticle formation. Here, the 60ZKB-600 sample shows ultrafine dispersion of Co@CoO_*x*_ nanoparticle over KB with particle size distribution between 3–7 nm. Compared to this, ZIF-600 appears to show some what aggregated nanoparticles. XPS survey and high-resolution Co 2p, O 1s and N 1s spectra and their deconvoluted components of 60ZKB-600 sample reveal relevant composition and chemical states of elements (Fig. 3e, f and S6†). The oxidation and reduction of Co^2+^ metal-centres along with carbonization and N-doping components from the decomposition of ZIF-67 can be seen.^8,15,22,23,27–33^ Co 2p spectra with two main peaks positioned at ∼780.3 and ∼795.8 eV can be assigned to the 2p_3/2_ and 2p_1/2_ doublets of Co^2+/3+^ structure, respectively. In agreement with Co@CoO_*x*_ (CoO and Co_3_O_4_) structure (Fig. 3e) and literature,^15,23,30–36^ the deconvoluted Co 2p_3/2_ spectra shows three characteristic peaks at ∼778.5, ∼780.0 and ∼781.4 eV and are relevant to metallic Co^0^, Co^3+^ and Co^2+^ components with the atomic contributions of about 12.5, 31.0 and 56.5%, respectively. Here, it is worth pointing that a large fraction of Co^2+^ component in the 60ZKB-600 sample supports for the co-existence of CoO and Co_3_O_4_ phases, evidenced in XRD data (Fig. 3a). Moreover, this Co^2+^ component also attribute to the typical Co-N_*x*_ coordination in the sample and agrees with the literature.^15,21–23,27–29^ The broad O 1s spectra with three prominent peaks further support this Co 2p spectral components (Fig. 3f). The deconvoluted three peaks in O 1s spectra accounts for about 22.5, 57.0 and 20.5 at% and are relevant to lattice oxygen (O_l_ or Co^2+^–O^2-^) of CoO_*x*_ phase at ∼529.8 eV, surface adsorbed oxygen (Co–OH/CO) at ∼531.3 eV and sp^3^ C–O at ∼533.0 eV, respectively. Overall, the XPS data suggests mildly oxidised and weakly crystalline Co/CoO_*x*_ nanoparticles embedded in N-doped carbon.^15,31–36^

All these results reveal the beneficial role of solution-grown ZIF-67 nanophase on the KB support and their thermally derived Co@CoO_*x*_ nanophase embedded in N–C matrix for efficient electrocatalytic ORR activity. Impressive *E*_onset_ and *E*_1/2_ values in thermolyzed 60ZKB-600 sample can be linked to the effective activation of adsorbates and fast electron transfer between active sites and adsorbates. Literature suggests that ultra-small amorphous/crystalline type Co@CoO_*x*_-based nanoparticles likely comprise rich exposed edges and defects/vacancies on the heterogeneous surface, which in turn helps to promote oxygen adsorption and catalysis reactions, under reduced reaction energy barriers.^15,23,30–36^ Furthermore, the nitrogen dopants in the carbon structures can improve the heterogeneity of the charged carbon surface due to the lone pair electron transfer from nitrogen to carbon. Thus, the dopants can create positive cores to enhance the oxygen binding.^23^ Metal or metal-oxide in the carbons further increases the electrostatic attraction of molecular oxygen. As discussed briefly, under the results of Fig. 1 and 2 data, the ZIF-67 derived single component Co–N–C and Co_3_O_4_–N–C structures under high temperatures show inferior ORR performance than 60ZKB-derived Co@CoO/Co_3_O_4_–N–C structure under mild temperature. This suggesting that a synergistic effect of improved electronic transfer and electrostatic interactions at the interface for the facile ORR reaction facilitated by Co(0)-phase combined with highly heterogeneous surface created by amorphous/crystalline CoO_*x*_-phase with exposed oxygen-rich vacancies.^15,27–36^ In fact, the literature confirms beneficial role of additional CoO phase in the Co/Co_3_O_4_–N–C structure with Co^2+^/Co^3+^ redox couple for promoting ORR performance.^29–36^

Finally, it is noted that the observed ORR for the Co@CoO_*x*_–N–C nanophases of thermolyzed 60ZKB-600 sample achieved under mild and simple synthesis conditions are competitive with the numerous Co/Co_3_O_4_ nanophase-based catalyst materials produced under complex design and high-temperature thermolysis routes (Table S2†).^1–34^ For example, *E*_onset_ = ∼0.90 V, *E*_1/2_ = ∼0.83 V and *J*_L_ = 4.0 mA cm^−2^ values in 60ZKB-600 sample are comparable to that of small bimetallic FeCo clusters in CNF (*E*_onset_ = 0.89 V, *E*_1/2_ = 0.78 V and *J*_L_ = 4.2 mA cm^−2^), even though this sample was obtained by electro-spun polyacrylonitrile (PAN) containing Fe and Co-nitrate salts in dimethylformamide following oxidation at 280 °C and then carbonization at 1000 °C.^2^ Likewise, bimetallic CoSe–N–C and Co@CoSe–N–C prepared by ZIF-67 mixed with Se and carbonized at 1000 °C had *E*_onset_ and *E*_1/2_ potentials of 0.88–0.90 and 0.80–0.82 V, respectively.^7^ In the work by Wan *et al.* CoNi–N–C derived from urea and butterfly wing biomass *via* hydrothermal reaction followed by carbonization at 800 °C exhibited inferior *E*_onset_, *E*_1/2_ and *J*_L_ values of 0.87 V, 0.8 V and 4.0 mA cm^−2^.^17^ Even the multi-metallic FeCoCu, FeCoZn and CoCuZn-based single-atom level M_1_–N–C catalysts showed comparable *E*_onset_ and *E*_1/2_ values to the 60ZKB-600; however, samples were produced *via* multi-step, by making M–C_3_N_4_ first (*via* MCl_2_ and melamine in HCl solution following pyrolysis of solids at 550 °C) and then carbonizing dopamine polymer grafted M–C_3_N_4_ at 800 °C.^6^ A similar extended synthesis route to generate single-atom based (Fe,Co)Se@Fe_1_–N–C catalysts also showed *E*_1/2_ values close to the ZKB-600 sample; in their synthesis, Fe_1_–N–C was obtained by pyrolysis of Fe-containing ZIF-8 at 900 °C followed by hydrothermal growth of FeCo double hydroxide on Fe_1_–N–C and further anion exchange at 160 °C and second carbonization at 500 °C.^3^ In another multicomponent manipulation, Co–N–C based catalysts, generated from carbonization of Co-anchored 2D lamellar poly (ionic liquid) (PIL) network embedded ZIF-67 at 700–900 °C under 5%H_2_/Ar atmosphere, also showed *E*_onset_, *E*_1/2_ and *J*_L_ values between 0.72 to 0.83 V, 0.61 to 0.76 V, and 3.2 to 4.2 mA cm^−2^, respectively; whereas *E*_onset_ = 0.81–0.88 V, *E*_1/2_ = 0.77–0.82 V and *J*_L_ = 2.5 to 4.1 mA cm^−2^ were observed in the Co-ZIF-8-derived Co–N–C/rGO based catalysts.^4^ The *E*_onset_, *E*_1/2_ and *J*_L_ values of 0.8–0.9 V, 0.75–0.83 V and ∼4.0 mA cm^−2^ from Co–N–C or Co@Co_3_O_4_–N–C structures deduced *via* pyrolyzed ZIF-67 following air oxidation are not better than the 60ZKB-600derived Co@CoO_*x*_–N–C performance reported in this work (Table S2†).

Therefore, all these results and discussion support the superior performance of Co@CoO_*x*_-nanoparticle embedded in N-doped KB support sample. Furthermore, it can be seen from the overall results, ZIF-67 : KB weight ratio is a prime influencing factor in enabling better ORR performance and when targeting lower thermolysis temperature for the synthesis of active cobalt-based amorphous/crystalline nanocrystals. This could be seen from the very first instance that compared to the thermolyzed ZIF-67 at 700 °C, which is poorly ORR active, 80ZKB hybrid treated under mild temperature of 600 °C showed significantly enhanced ORR performance. This observation encouraged further to optimize the composition of *x*ZKB samples to achieve acceptable level ORR performance *via* low-temperature thermolysis route.

## Conclusions

This work reports a facile fabrication route to ultrafine Co@CoO_*x*_ nanoparticles dispersed on N-doped commercial carbon black (Ketjenblack–KB) that acts as a high-performance ORR electrocatalyst. For this, scalable ZIF-67 nanocrystals were solution grown on the as-purchased KB at room temperature following a mild carbothermal optimization. Here, tuned ZIF-67 : KB weight ratio facilitated for synthesis of electrocatalytically active cobalt-based nanocrystals under low-temperature modulation and for better ORR activity. The ORR performance in 0.1 M KOH in a typical RDE setup delivered LSV polarization curves with high positive onset and half-wave potentials close to 0.9 V and 0.83 V, respectively. These values are comparable to peer metal-nitrogen-carbon based electrocatalysts; however, they were designed under extensive physicochemical modulations of multi-component precursors and under relatively high thermolysis conditions. The selection of widely available low-cost carbon black, scalable ZIF-67 crystal growth under ambient conditions and mild thermal modulation applied in this work could be promising route for the development of electrocatalysts, that also utilize dimensionally and chemically variable MOF-structures, and could be applied for various applications, including oxygen evolution and carbon dioxide and nitrogen reduction reactions (OER, CO_2_RR and NRR, respectively).

## Conflicts of interest

There are no conflicts to declare.

## Supplementary Material

RA-013-D3RA07754C-s001
