# Supplementary material for: Efficient electrocatalytic oxygen reduction reaction of thermally optimized carbon black supported zeolitic imidazolate framework nanocrystals under low-temperature
Source: RSC Adv. 2023 Nov 24;13(49):34556–61. doi: 10.1039/d3ra07754c (PMC10668571; doi:10.1039/d3ra07754c)
Supplement: RA-013-D3RA07754C-s001 [file RA-013-D3RA07754C-s001.pdf]

Electronic Supplementary Material (ESI) for RSC Advances.
This journal is © The Royal Society of Chemistry 2023

Efficient electrocatalytic oxygen reduction reaction of thermally optimized carbon black supported zeolitic imidazolate framework nanocrystals under low-temperature

Jinyi Chen,[1] Jian Guo,[1] Hong Zhang,[1] Dan J. L. Brett,[2] and Srinivas Gadipelli,*[,1,2]

[1]College of Physics, Sichuan University, Chengdu, 610064 China
[2]Electrochemical Innovation Lab, Department of Chemical Engineering, University College London, London, WC1E 7JE, UK
*Correspondence: s.gadipelli@ucl.ac.uk

Experimental details

Materials

The following materials were purchased and used as-received. Ketjenblack-EC300J(KB), 2-4 mm (Sinero); cobalt(II) nitrate hexahydrate ($Co(NO_3)_2{\cdot}6H_2O$), 98% (Sigma-Aldrich); 2-methylimidazole (2MIM; $CH_3C_3H_2N_2H$), 99% (Acros Organics); triethylamine (TEA; $N(CH_2CH_3)_3$), ≥99.5% (Sigma-Aldrich); polyvinylpyrrolidone (PVP; $(C_6H_9NO)_n$), average molecular weight 4000 (Sigma-Aldrich); methanol (CH_3OH), ≥99.5% (Chengdu Changlian); potassium hydroxide (KOH), ≥85% pellets (Alfa Aesar); Nafion ($C_7HF_{13}O_5S{\cdot}C_2F_4$) solution, 5 wt% (Sigma-Aldrich).

Synthesis of ZIF-67

$Co(NO_3)_2{\cdot}6H_2O$ (5.9 g) and PVP (5 g) were dissolved in 500 mL of methanol under stirring for 10 minutes to form a solution A. 2MIM (6.65 g) and TEA (600 µL) were dissolved in another 500 mL of methanol to form solution B. Then, solution A was slowly added to solution B under stirring for a few hours and left undisturbed overnight. The mother liquor decanted and settled solid product was washed with fresh methanol by centrifugation. Finally, the sample was dried at 60 °C in an oven and used for further experiments.

Synthesis of 80ZKB

This sample is a ZIF-67:KB in about 80:20 weight ratio, abbreviated as 80ZKB. This sample was synthesised according to the literature.[15] $Co(NO_3)_2{\cdot}6H_2O$ (4800 mg) and PVP (4086 mg) were dissolved in 408 mL of methanol to form a solution A. Finely ground KB (300 mg) in agate mortar was dispersed in 408 mL of methanol by sonication for 30 minutes and 2MIM (5415 mg) and TEA (489 µL) were dissolved to form solution B. Solution A was then slowly added dropwise to solution B with continuous stirring, and the mixture was allowed to continue stirring for 8 hours and left setting overnight. The product was washed with fresh methanol and collected by centrifugation. Finally, the sample was dried at 60 °C in an oven and used as such for further experiments.

The weight percentage of ZIF-67 grown on the surface of KB was calculated by comparing the final product weight (W1) of ZKB and the original KB weight (W2), the weight percentage (wt%) of ZIF-67 in the sample = (W1 – W2) x 100/W1.

The other composition ZKB samples, with about 60, 40 and 20 wt% of ZIF-67 in ZKB (named 60ZKB, 40ZKB and 20ZKB), were synthesized using the same procedure as above, by changing the quantity of precursors and solvent used as listed in the **Table S1** below.

Table S1. Precursor and solution amounts for synthesis of 80ZKB, 60ZKB, 40ZKB and 20ZKB samples

	A solution			B solution			
	$Co(NO_3)_2{\cdot}6H_2O$ (mg)	PVP (mg)	CH_3OH (ml)	KB (mg)	2MIM (mg)	TEA (µL)	CH_3OH (ml)
80ZKB	4800	4086	408	300	5415	489	408
60ZKB	1800	1532	153	300	2031	184	153
40ZKB	800	681	68	300	903	82	68
20ZKB	300	256	26	300	338	31	26

Thermolysis of ZIF-67, and 80ZKB, 60ZKB, 40ZKB and 20ZKB samples

Typically, 30-50 mg of ZIF-67 and/or ZKB samples each in alumina boats were placed into a horizontal tube furnace (Zhengzhou TCH instrument Co., Ltd.), which was Ar purged at room temperature, and thermolysis of the samples was carried out for 6 hours at a given temperature of 500 or 600 or 700 °C under a continuous flow of argon gas at a heating rate of 5 per minute. The thermolyzed ZIF-67 samples at 500, 600 and 700 °C were named as ZIF-500, 600 and ZIF-700, respectively. Likewise, thermolyzed 80ZKB samples at 500, 600 and 700 °C were named as 80ZKB-500, 80ZKB-600 and 80ZKB-700, respectively. The same notation applied for thermolyzed 60ZKB, 40ZKB and 20ZKB samples.

Characterization

Powder XRD (Co Kα radiation, Panalytical) was carried out in the scan range of 2θ = (2-80)° and step size of 0.013°. X-ray photoelectron spectroscopy (XPS, AXI, Supra, Kratos) data, scanning electron microscopy (SEM, Aztec Live ULTIM) and transmission electron microscopy (TEM, JEM-2100Plus) measurements were carried out on the samples supported on a carbon tape or a carbon-coated copper TEM grid.

Electrochemical measurements

The electrochemical measurements were conducted using a Bio-Logic electrochemical workstation (DHS Instruments Company) with a standard three-electrode system in O_2-purged 0.1 M KOH electrolyte. Ag/AgCl (in a saturated KCl aqueous solution) and Pt-wire served as reference and counter electrodes, respectively. The working electrode was a glassy carbon-based rotating disk electrode (RDE, Φ = 5 mm, area = 0.19625 cm^2). For the electrochemical tests, the catalyst was prepared as follows: 5.00 mg powder sample was dispersed in 1 mL Nafion/deionized water (40 µL/960 µL) solution via sonicate for about 30 to 45 minutes to form a homogeneous ink. Then, 10.0 µL as-prepared catalyst ink was pipetted and drop-casted onto the polished clean and dry RDE and allowed to dry at 45 °C for about half an hour. Catalyst loading per unit electrode area of all the samples was about 0.254 mg cm^{-2}. The higher catalyst loadings of 0.318 and 0.382 mg cm^{-2} were obtained by increasing the drop-casting ink volume to 12.5 and 15.0 µL, respectively. Cyclic voltammetry (CV) curves were measured at a scan rate of 50 mV s^{-1} initially until stable overlapping CV curves were obtained (which requires 20 to 50 scans) and actual data was recorded at a scan rate of 10 mV s^{-1} in the potential range of +0.2 V to -0.8 V. Linear sweep voltammetry (LSV) polarizations were recorded at a scan rate of 10 mV s^{-1} in the same potential range of +0.2 V to -0.8 V. ORR LSV curves were measured at rotational speed of 1600 rpm. The measured potentials (*vs*. Ag/AgCl) were converted to be relative to the reversible hydrogen electrode (RHE) using the equation: V vs. (RHE) = V vs. (Ag/AgCl) + 0.059 × pH +0.197.

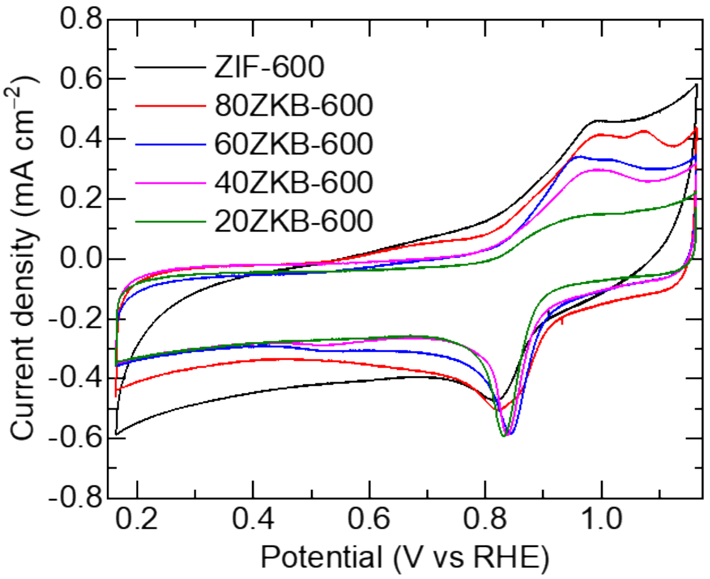

Figure S1. CV curves of xZKB-600 and ZIF-600 samples, measured in 0.1 M KOH at 10 mV s^{-1} scan rate.

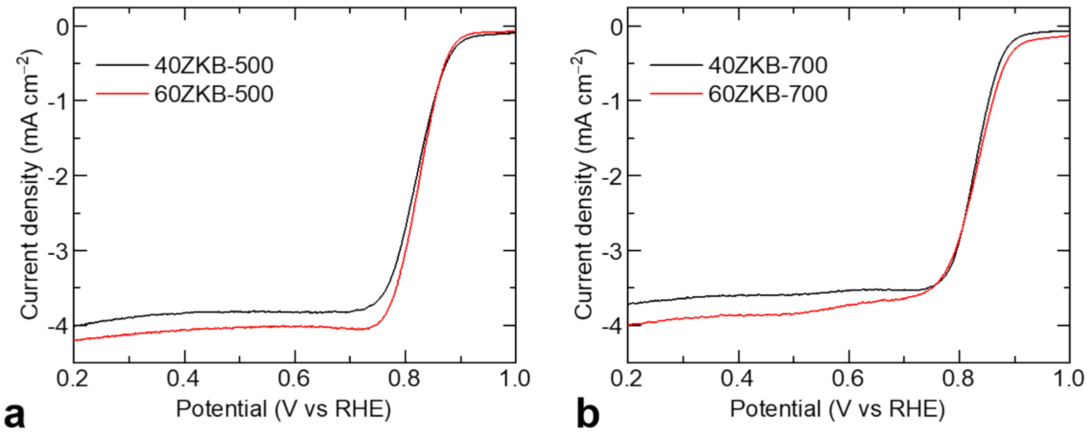

Figure S2. Comparative LSV curves of 40ZKB and 60ZKB samples thermolyzed at a) 500 °C and b) 700 °C. This shows better ORR performance of thermolyzed 60ZKB samples over thermolyzed 40ZKB samples.

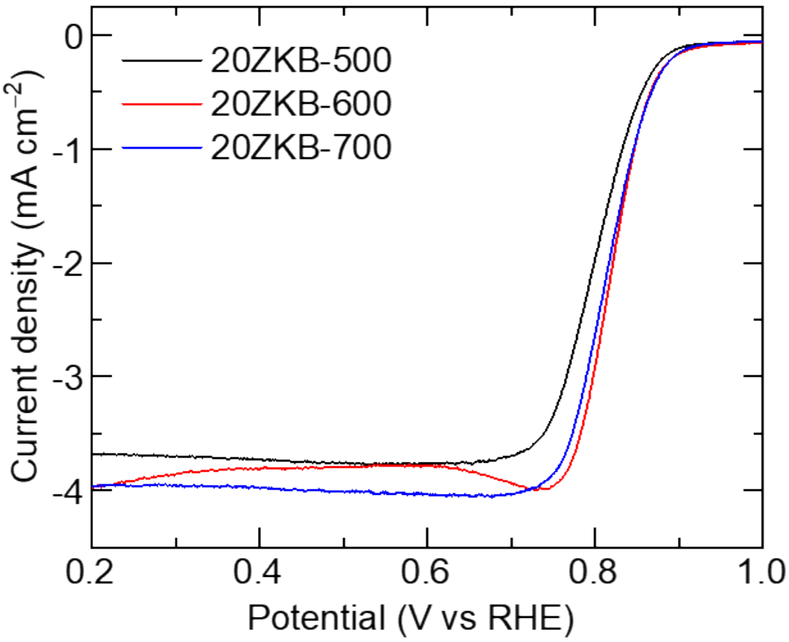

Figure S3. Comparative LSV curves of 20ZKB samples thermolyzed at 500, 600 and 700 °C; Among these, 20ZKB-600 shows better ORR performance.

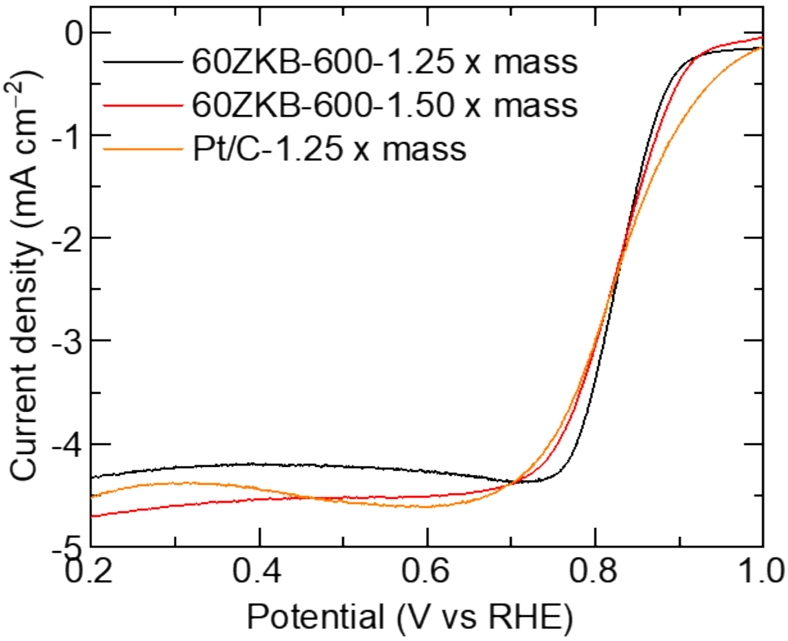

Figure S4. LSV curves of 60ZKB-600 and Pt/C samples measured with higher catalyst loadings of 0.318 mg cm^{-2} (noted as 1.25 x mass) and 0.382 mg cm^{-2} (noted as 1.50 x mass), which were obtained by increasing drop-casting catalyst ink volume to 12.5 and 15.0 µL, respectively, compared to 0.254 mg cm^{-2} loading from 10.0 µL volume of catalyst ink in all other tested samples. This data shows an improved limiting current density compared to the data showed in Figures 2 and Figures S2-S3.

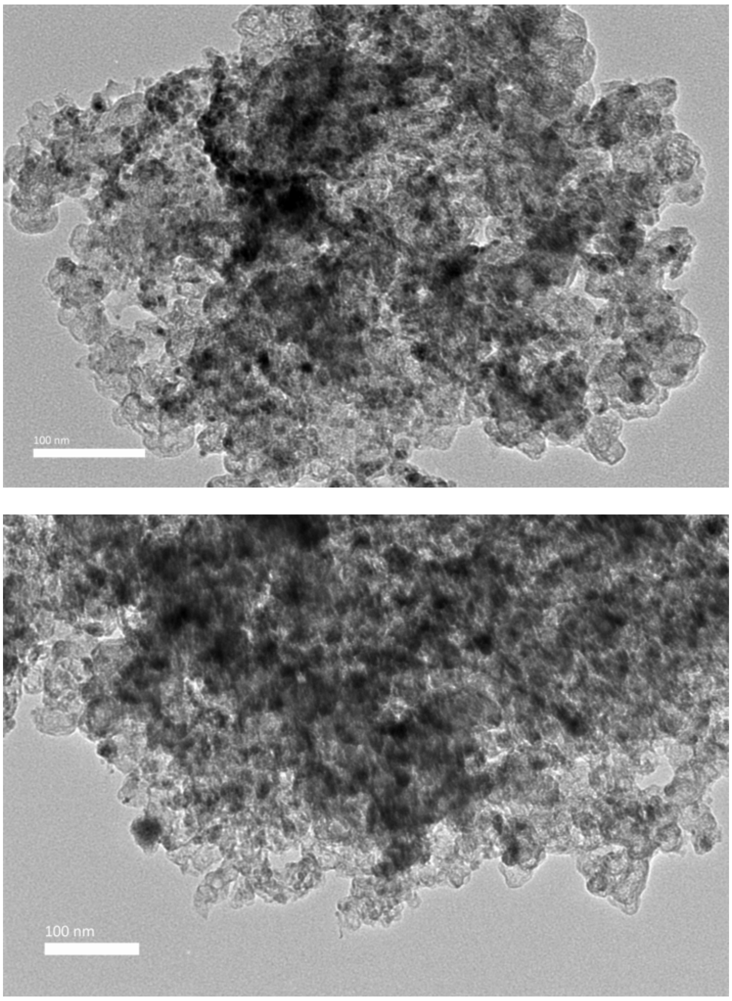

Figure S5. TEM micrographs of 60ZKB-600 sample.

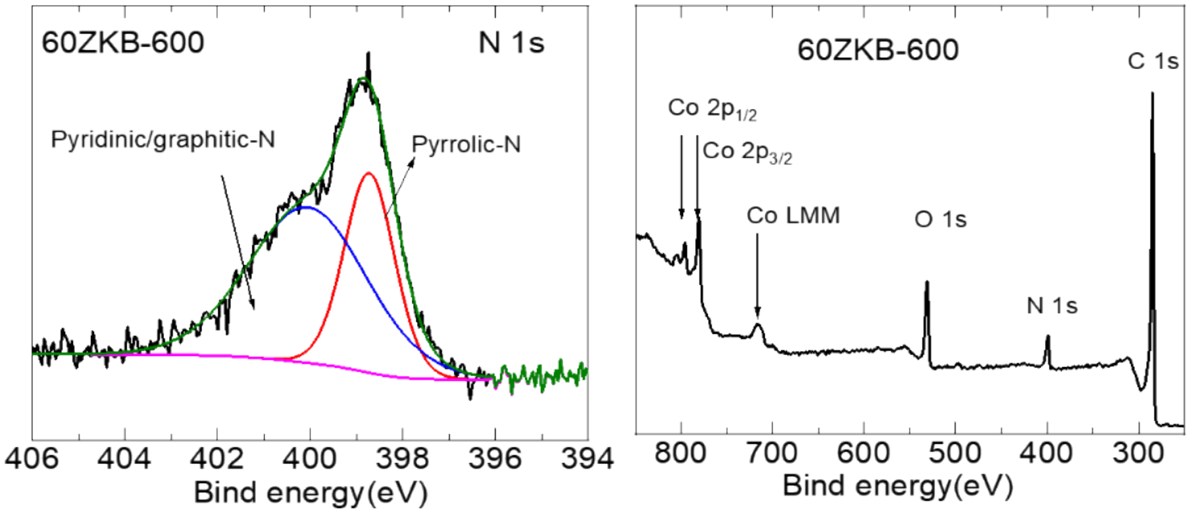

Figure S6. High-resolution XPS N 1s spectra with deconvoluted peak fittings (left) and overall survey spectra (right) for 60ZKB-600 sample. The conversion of ligand pyrrolic-N component to pyridinic-N (at ~398.7 eV) and combined pyrrolic/graphitic-N (~400 eV) can be seen (see main ref. 8,15,22,23). The overall survey spectra account for about 82.7 at% C, 9.27 at% O, 5.72 at% N and 2.46 at% Co in the sample.

Table S2. Literature reported M@MO$_x$-N-C based catalysts with carbonization temperature and ORR performance E_{onset}, $E_{1/2}$ and J_L values in a 0.1 M KOH electrolyte.

	Sample	E_{onset} (V)	$E_{1/2}$ (V)	J_L (mA cm^{-2})	Ref.
1	**60%ZKB-600**	**0.90**	**0.83**	**4.0**	**This work**
2	(Fe,Co)Se$_2$+Fe$_1$/NC<br><br>Derived from Fe-ZIF-8 at 900 °C followed by hydrothermal FeCo double hydroxide on Fe$_1$/NC and anion exchange to selenides at 160 °C and thermolysis at 500 °C.	0.93	0.836	4.0	3
3	Co-PIL-700-900<br><br>Co-ZIF-8-PIL-700-900 or Co-N-C/rGO<br><br>Derived from Co-anchored 2D lamellar poly(ionic liquid) (PIL) or rGO network embedded ZIF-67 and Co-ZIF-8 following pyrolysis at 700-900 °C under 5%H$_2$/Ar atmosphere.	0.72-0.83<br><br>0.81-0.88	0.61-0.76<br><br>0.77-0.82	3.2-4.2<br><br>2.5-4.1	4
4	Mn-N-C and Co-N-C<br><br>Produced by pyrolyzing Mn-ZIF-8 and Co-ZIF-8 at 950 °C and was treated with a 10% H_2SO_4 solution at 70 °C for 16 h.	0.90	0.82-0.83	3.2-3.5	5
5	Co@NC<br><br>From pyrolyzed ZIF-67 at 1000 °C	0.88	0.81	3.9	7
6	Co-N-CNT<br><br>Made from pyrolyzing Co-melamine coated oxidized CNT at 800 °C	0.88	0.81	4.3	9
7	Co nanorods@Co-N-C supported by carbon felt (CF)<br><br>Derived from pyrolyzed ZIF-67@CF	0.82	0.70	3.5	10
8	Co_3O_4-N-C<br><br>Derived from carbonized cobalt-phthalocyanine ($C_{32}H_{16}CoN_8$) with NaCl and KCl solution at temperatures between 700-1000 °C	0.85	0.77	3.9	11
9	Co-N-C<br><br>Derived from pyrolyzed Co-MOF nanosheets at 500-700 °C	0.78-0.86	0.62-0.78	2.9-4.0	12
10	Co@Co_3O_4-N-C	0.88	0.82	4.4	13

	Derived pyrolyzed from ZIF-L@CFP (CFP was oxidized in conc. HNO_3 in an autoclave at 100 °C) at 700 °C				
11	Co-N-C; Ni-N-C and CoNi-N-C<br><br>Derived from urea and butterfly wing via hydrothermal and carbonization at 800 °C	0.84-0.87	0.72-0.80	2.8-4.0	17
12	Co_3O_4@N-CNFs<br><br>Derived via pyrolyzed electro-spun PAN fibers at 800 °C followed by Co_3O_4 growth by atomic layer deposition	0.87	0.70	3.8	19
13	Ni-N-C<br><br>Derived from pyrolyzed Ni-ZIF-8 at 800-1100 °C	0.80-0.89	0.62-0.75	3.3-4.0	22
14	Co-N-C<br><br>Derived from ZIF-67 at 1000 °C	0.86	0.78	4.2	23
15	Co-N-CNT<br><br>Made from pyrolyzing Co-melamine coated oxidized CNT at 800 °C	0.87	0.79	3.6	27
16	Co-N-C and FeCo-N-C<br><br>Derived from pyrolyzed Fe-ZIF-67+dicyandiamide at 800 °C	0.87	0.79-0.80	3.4-5.5	28
17	Co-N-C<br><br>Derived from pyrolyzed Co-phenanthrolyne-graphene oxide at 1000 °C	0.87	0.79	4.2	29
18	Co-N-C<br><br>Derived from the pyrolyzed ZIF-67 at 800 °C	0.88	0.70	3.8	30
19	CoO_x-N-C<br><br>Derived from pyrolyzed ZIF-67@rGO	0.83-0.88	0.77-0.80	3.8-4.6	31
20	CoO-N-C<br><br>Derived from pyrolyzed ZIF-67 with carbon powder at 750 °C	0.86-0.89	0.81-0.83	~5.0	32
21	Co@CoO-N-C<br><br>Derived from pyrolyzed ZIF-67 at 700 °C	0.90	0.84	4.30	33
22	Co@Co_3O_4/NC<br><br>Air oxidation of carbonized ZIF-67 at 800 °C	0.88	0.74	4.2	34
23	Cu_1–N–C<br><br>Made from Cu^{2+}-polydopamine coated	0.88	0.80	3.90	S1

	diblock copolymer poly(ethylene oxide)-block-polystyrene template carbonized in a NH_3 atmosphere at 700 °C.				
24	Co@N-C and acid treated Co@N-C<br><br>Derived from pyrolyzed $Co_3[Co(CN)_6]_2$ Prussian blue MOF at 400-900 °C	0.90	0.83	3.7-4.0	S2
25	Co@NC and Co@Co_3O_4@NC<br><br>Oxidation of pyrolyzed Co-polydopamine at 900 °C	0.88-0.90	0.76-0.78	4.0-4.2	S3
26	Co–CeO_2–N–C derived from carbonized electrospun PAN nanofibers at 900 °C	0.87	0.82	3.8	S4
27	Co_xNi_y@NC<br><br>From carbonized CoNi-dicyanodiamide at 800 °C	0.77-0.87	0.70-0.76	2.8-4.0	S5

References

S1. Qi, C., Yang, H., Sun, Z., Wang, H., Xu, N., Zhu, G., Wang, L., Jiang, W., Yu, X., Li, X., Xiao, Q., Qiu, P., Luo, W., Modulating Electronic Structures of Iron Clusters through Orbital Rehybridization by Adjacent Single Copper Sites for Efficient Oxygen Reduction, *Angew. Chem. Int. Ed.* 2023, 62, e202308344.

S2. Zeng, M., Liu, Y., Zhao, F., Nie, K., Han, N., Wang, X., Huang, W., Song, X., Zhong, J. and Li, Y. Metallic Cobalt Nanoparticles Encapsulated in Nitrogen-Enriched Graphene Shells: Its Bifunctional Electrocatalysis and Application in Zinc–Air Batteries. *Adv Funct Materials* **26**, 4397–4404 (2016).

S3. Wang, Z., Li, B., Ge, X., Goh, F.W.T., Zhang, X., Du, G., Wuu, D., Liu, Z., Andy Hor, T.S., Zhang, H. and Zong, Y. Co@Co_3O_4@PPD Core@bishell Nanoparticle-Based Composite as an Efficient Electrocatalyst for Oxygen Reduction Reaction. *Small* **12**, 2580–2587 (2016).

S4. Zhang, Z., Gao, D., Xue, D., Liu, Y., Liu, P., Zhang, J., and Qian, J., Co and CeO_2 co-decorated N-doping carbon nanofibers for rechargeable Zn–air batteries, *Nanotechnology,* 2019, **30**, 395401.

S5. Ran, J., Guo, X., Liu, P., Peng, S., Gao, X., and Gao, D., Bifunctional catalysts of CoNi nanoparticle-embedded nitrogen-doped carbon nanotubes for rechargeable Zn–air batteries, *Nanotechnology,* 2019, **30**, 435701.
